# The diagnostic value of two antigenic domains derived from the *Trypanosoma cruzi* Tc323 protein in chronic Chagas disease: a study in Brazil

**DOI:** 10.3389/fmicb.2025.1605755

**Published:** 2025-06-10

**Authors:** Micaela S. Ossowski, Ângelo Antônio Oliveira Silva, Emily Ferreira Santos, Leonardo Maia Leony, Fred Luciano Neves Santos, Karina A. Gómez

**Affiliations:** ^1^Instituto de Investigaciones en Ingeniería Genética y Biología Molecular “Dr. Héctor N. Torres” (INGEBI-CONICET), Buenos Aires, Argentina; ^2^Advanced Health Public Laboratory, Gonçalo Moniz Institute, Oswaldo Cruz Foundation (FIOCRUZ-BA), Salvador, Brazil; ^3^Interdisciplinary Research Group in Biotechnology and Epidemiology of Infectious Diseases (GRUPIBE), Gonçalo Moniz Institute, Oswaldo Cruz Foundation (FIOCRUZ-BA), Salvador, Brazil; ^4^Integrated Translational Program in Chagas Disease from Fiocruz (Fio-Chagas), Rio de Janeiro, Brazil

**Keywords:** *Trypanosoma cruzi*, chronic Chagas disease, Tc323, diagnosis, ELISA

## Abstract

The diagnosis of chronic infection with the parasite *Trypanosoma cruzi* relies on detecting specific IgG antibodies using two or three (in the case of discordance) serological assays based on different principles. This diagnostic algorithm poses challenges in identifying patients with chronic Chagas disease (CCD), particularly in endemic areas where financial and human resources are scarce. The discovery of new antigens capable of achieving 100% sensitivity and specificity is a priority in this field. Previously, we introduced two recombinant domains, designated as rTcD3 and rTcD6 derived from a hypothetical protein from *T. cruzi*, as potential candidates for the diagnosis of CCD. In the current study, we extended our results by assessing the diagnostic accuracy of rTcD3 and rTcD6 in a large cohort of infected and non-infected individuals from various regions of Brazil. Both antigens showed a specificity of 97.9%, while sensitivity was 79.5% for rTcD3 and 81.5% for rTcD6. In addition, cross-reactivity analysis on 764 samples from individuals with other parasitic, bacterial and viral infections was estimated to be less than 0.9%. Specifically, one (for rTcD3) and two (for rTcD6) samples positive for leptospirosis reacted with both antigens, while 2 out of 764 samples from individuals infected with *Leishmania* spp., resulted in a false positive for rTcD3, while four samples behaved similarly for rTcD6. Furthermore, a false-positive reaction was also observed in one HIV-positive sample rTcD6. In conclusion, this study provides further evidence supporting the diagnostic potential of a specific and sensitive IgG-ELISA based on rTcD3 and rTcD6 for detecting chronic *T. cruzi* infection across various regions of the Americas, with minimal cross-reactivity with other pathogens.

## 1 Introduction

Chagas disease (CD), caused by the protozoan parasite *Trypanosoma cruzi*, remains a significant public health challenge, primarily in Latin America but increasingly recognized worldwide due to migration. The disease progresses from an acute phase to a chronic phase, often remaining asymptomatic for decades before causing severe cardiac, digestive, or neurological complications (WHO, 2020).

Early and accurate diagnosis is crucial for effective management, epidemiological surveillance, and transmission prevention, particularly in endemic regions. The chronic phase of Chagas disease (CCD) poses significant diagnostic challenges due to low parasitemia levels, demanding the use of serological methods to detect specific antibodies against *T. cruzi*. Commonly employed assays include enzyme-linked immunosorbent assay (ELISA), indirect immunofluorescence (IIF), and indirect hemagglutination (IHA) (Schijman et al., 2024). However, these methods are prone to false positives and inter-assay variability. To mitigate these issues, the World Health Organization (WHO) recommends using two concordant serological tests, with a third test performed in case of discordant results, to improve diagnostic accuracy.

The lack of a single assay with 100% sensitivity and specificity demands resources, infrastructure, and trained personnel that are often unavailable in regions where the disease is endemic. Beyond these logistical constraints, biological aspects such as cross-reactivity with antibodies from other parasitic infections and the genetic diversity of *T. cruzi* further complicate the diagnosis. The parasite’s extensive genetic variability is classified into six discrete typing units (DTUs), TcI–TcVI, and an additional genotype, Tcbat (Zingales et al., 2009, 2012). This diversity correlates with geographical distribution, eco-epidemiological traits, and disease progression (Silvestrini et al., 2024). For instance, while TcI is widely dispersed across the Americas, TcV and TcIV are predominantly found in southern and central South America. Consequently, serological tests developed with antigens from specific regions may underperform in detecting infections caused by strains from other areas (Truyens et al., 2021). Furthermore, the host’s antibody response is influenced by the antigenic variability of *T. cruzi* strains and the host’s genetic background (Silvestrini et al., 2024), directly impacting diagnostic sensitivity and specificity.

In recent work, we demonstrated the diagnostic accuracy of two immunogenic domains derived from the hypothetical *T. cruzi* protein Tc323, evaluated using an *in-house* ELISA. A panel of 333 CCD-positive and 141 CCD-negative plasma/serum samples from seven endemic countries (Argentina, Bolivia, Colombia, Mexico, Paraguay, El Salvador, and the USA) was tested. The recombinant domain rTcD3 achieved a sensitivity of 90.7% and specificity of 92.2%, while rTcD6 exhibited a sensitivity and specificity of 93.1 and 93.6%, respectively. Additionally, cross-reactivity was assessed using samples from individuals with related infectious (cutaneous leishmaniasis, strongyloidiasis, and toxoplasmosis), yielding false-positive results in 5/49 and 4/49 samples for rTcD3 and rTcD6, respectively (Ossowski et al., 2024).

In this study, we expanded our investigation of the diagnostic performance of the recombinant proteins rTcD3 and rTcD6 in distinguishing *T. cruzi*-positive and -negative individuals with CCD. To do this, we analyzed a panel of serum samples collected in Brazil, which includes both endemic regions (Bahia, Goiás, Minas Gerais, and Pernambuco) and a non-endemic region (Paraná). Notably, these regions exhibit a distinct distribution of *T. cruzi* discrete typing units (DTUs) compared to those evaluated in our previous study (Zingales and Macedo, 2023). Furthermore, we aimed to include not only a larger panel of sera from individuals with other infectious diseases, but also to broaden the range of pathogens represented—such as viruses and bacteria—in order to more comprehensively evaluate the potential cross-reactivity of the recombinant antigens.

## 2 Materials and methods

### 2.1 Sampling

**Figure 1:**
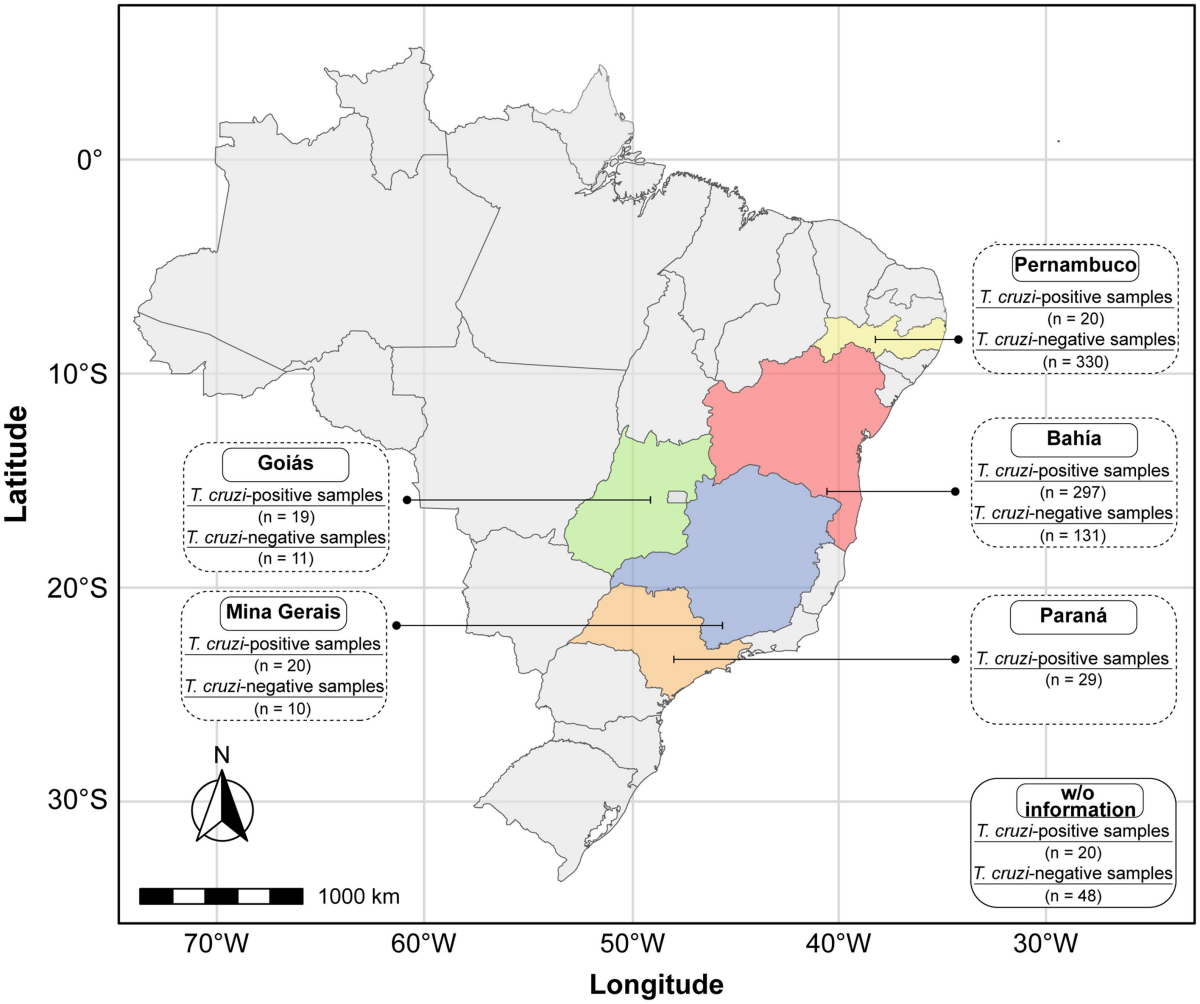
Regional distribution of the samples in Brazil. *T. cruzi-*positive and negative samples were collected from both endemic and non-endemic areas. The box positioned in the lower right corner denotes the number of patients with CCD and non-infected donors for whom origin data were unavailable.

The sample size was calculated using R software (4.3.2) (R Core Team, 2023), employing the pwr.r.test function from the pwr package (Champley, 2020). Assuming a 95% confidence interval, expected sensitivity and specificity of 95%, and an allowable error margin of 2%, the minimum required sample size was determined to be 316 sera from *T. cruzi*-positive and 316 negative donors. For this study, a total of 405 sera from individuals with CCD and 530 sera from non-infected donors were collected from both endemic and non-endemic regions of Brazil, including Bahia (BA), Minas Gerais (MG), Goiás (GO), Paraná (PR), and Pernambuco (PE). The geographical distribution of these samples is shown in Figure 1, generated using the *rnaturalearth* package in RStudio, exported as an SVG file (Massicotte and South, 2024), and refined using Adobe Illustrator. A summary of the number of samples with unavailable origin data is presented in the lower-right inset of the Figure. To ensure the reliability of our study, all samples were obtained from local reference laboratories (Central Public Health Laboratories; LACENs) and had undergone prior characterization for the presence of anti-*T. cruzi* antibodies. This characterization involved the use of two serological assays, performed according to internationally recognized guidelines for Chagas disease diagnosis (Pan American Health Organization, 2019).

Additionally, the study included 764 sera from individuals with unrelated diseases confirmed by serological or parasitological diagnosis. These diseases included: COVID-19 (*n* = 10), dengue (*n* = 21), schistosomiasis (*n* = 45), filariasis (*n* = 50), hepatitis B (*n* = 78), hepatitis C (*n* = 49), HIV (*n* = 76), HTLV (*n* = 43), cutaneous leishmaniasis (*n* = 65), visceral leishmaniasis (*n* = 89), leptospirosis (*n* = 97), leprosy (*n* = 15), rubella (*n* = 16), and syphilis (*n* = 110). All serum samples were re-evaluated for *T. cruzi* antibodies using serological assays. Samples with discordant or inconclusive results were excluded from this study.

This study was conducted in accordance with the Declaration of Helsinki and approved by the Institutional Review Board (IRB) for Human Research at the Gonçalo Moniz Institute (IGM), Oswaldo Cruz Foundation (FIOCRUZ), Salvador, Bahia (BA), Brazil (protocol no. 67809417.0.0000.0040). Written informed consent was obtained from all participants prior to sample collection.

### 2.2 Heterologous expression and purification of rTcD3 and rTcD6 proteins

The expression of rTcD3 and rTcD6 was performed as described by Ossowski et al. (2024). Briefly, *Escherichia coli* BL21 (DE3) pLysS cells were transformed with the recombinant expression vectors pRSET-A-6 × His-TcD3 or pRSET-A-6 × His-TcD6. Transformed bacteria were cultured in Luria-Bertani medium supplemented with 100 μg/μL of ampicillin-chloramphenicol and induced with 1 mM of Isopropyl *β*-D-1-thiogalactopyranoside (IPTG, Sigma-Aldrich, USA). Induction was carried out overnight at 21°C for rTcD3 and for 3 h at 37°C for rTcD6. Proteins were purified from inclusion body pellets by affinity chromatography and analyzed by SDS-PAGE and Western Blot (Ossowski et al., 2024) rTCD3 and rTCD6 concentrations were determined using Bradford assays (Bradford, 1976).

### 2.3 Indirect ELISA

The diagnostic performance of rTcD3 and rTcD6 was evaluated using an *in-house* ELISA protocol, modified from Ossowski et al. (2024). Following checkerboard titration, 96-well microplates (Greiner Bio-One GmbH, Germany) were coated with 10 ng/well of rTcD3 or rTcD6 in 100 μL of carbonate–bicarbonate buffer (0.05 M, pH 9.6) at room temperature for 15 min. Wells were blocked with 100 μL of WellChampion™ (Ken-En-Tec Diagnostics A/S, Denmark) according to the manufacturer’s instructions, then dried at 37°C for 90 min. Plates were washed five times with 250 μL of PBS-T buffer (PBS containing 0.05% Tween-20, pH 7.2). Serum samples diluted 1:100 in 100 μL of PBS-T were incubated for 1 h at 37°C. Following washing, 100 μL of horseradish peroxidase (HRP)-conjugated secondary antibody (SIGMA A8792; Sigma-Aldrich, USA) diluted 1:80,000 in PBS-T was added for 30 min at 37°C. After additional washing, immune complexes were visualized using 100 μL of TBM PLUS2 substrate (Kem-En-Tec, Denmark), with reaction halted after 15 min by adding 50 μL of 0.3 M H_2_SO_4_. Optical density (OD) was measured at 450 nm using a SPECTRAmax 340PC1 microplate reader (Molecular Devices, USA).

### 2.4 Data analysis

Data was processed in Microsoft Excel (2019, Microsoft Corp., USA) and analyzed using R software (4.3.2) (R Core Team, 2023). All analyses were two-tailed, with statistical significance defined as *p* < 0.05. To ensure the reliability and reproducibility of our ELISA experiments, we included internal controls on each microplate. Specifically, ten *T. cruzi*-positive and ten *T. cruzi*-negative samples, previously characterized as positive or negative based on two serological tests following international guidelines, were assessed simultaneously in all microplates. These samples were used to establish the relevant cutoff values (CO) for the immunoassays. Results were normalized as reactivity index (RI), calculated as the signal-to-cutoff ratio of the OD values, with RI > 1.00 considered positive. Samples within the gray zone of 1.0 ± 10% RI were classified as inconclusive. Descriptive statistics were presented as medians and interquartile ranges (IQR). Data normality was assessed using the Shapiro–Wilk test, while homogeneity of variance was tested using the Levene test. Non-parametric analyses were performed using the Wilcoxon-Mann–Whitney test for two groups and the Kruskal-Wallis test for three or more groups. Statistical significance was defined as *p* < 0.05.

The *pROC* package (Turck et al., 2011) was employed to estimate, compare, and apply the area under the curve (AUC) to determine optimal assay cut-offs. Diagnostic performance was assessed by calculating sensitivity (SEN), specificity (SPE), accuracy (ACC), likelihood ratios (LR), and diagnostic odds ratio (DOR), with 95% confidence intervals (CI). Agreement was evaluated using Cohen’s Kappa statistic with the *KappaGUI* package (Cohen, 1960; Santos, 2022).

**Figure 2:**
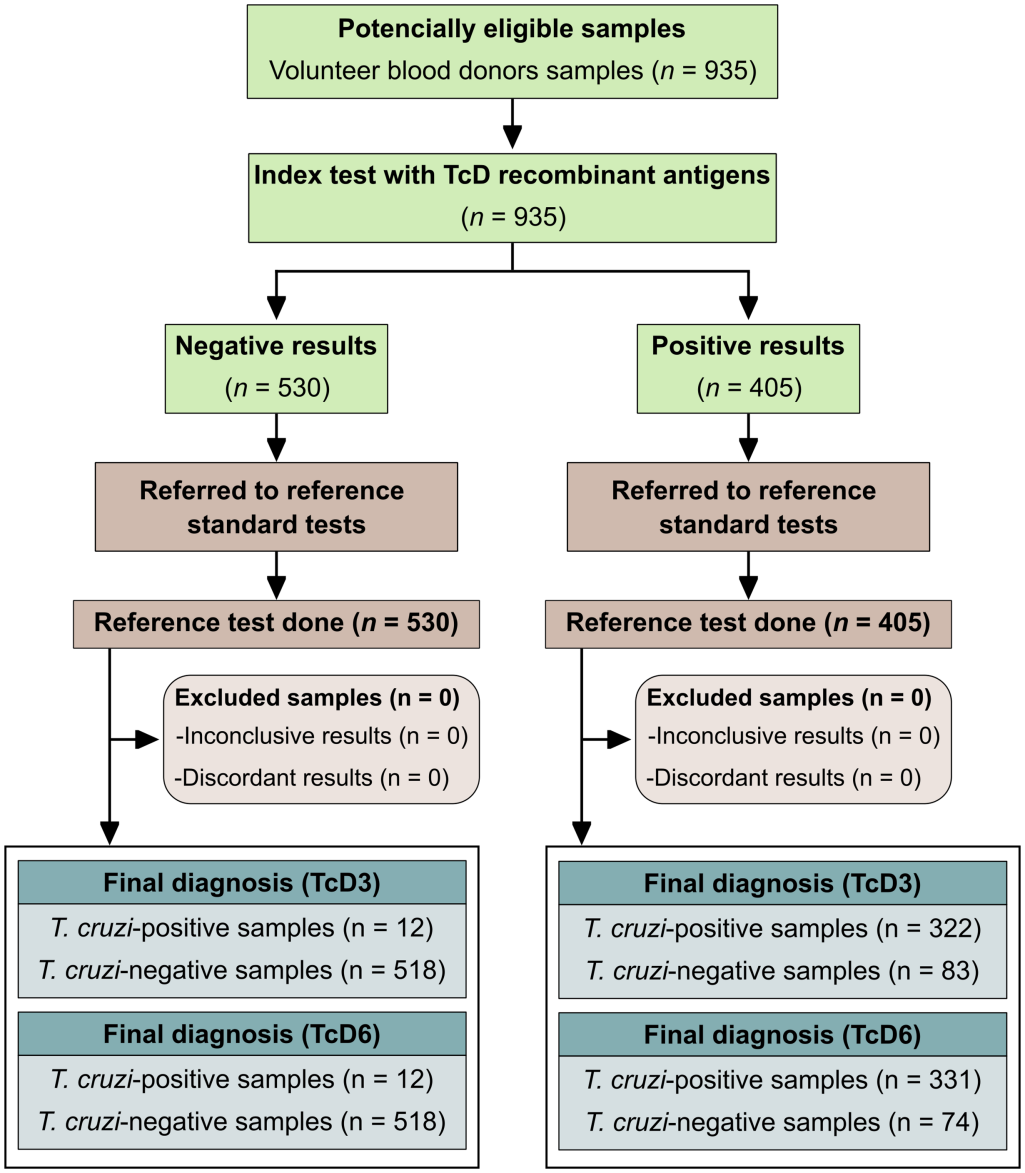
Flowchart depicts the study design following the Standards for Reporting of Diagnostic Accuracy Studies (STARD) guidelines.

A checklist (S1 Checklist) and flowchart (Figure 2) were included following the Standards for Reporting Diagnostic Accuracy Studies (STARD) guidelines (Cohen et al., 2016).

## 3 Results

### 3.1 Samples characterization

A total of 405 sera from individuals with CCD and 530 sera from non-infected donors were analyzed. These samples were collected from both endemic (Bahia, Minas Gerais, Goiás, and Pernambuco) and non-endemic (Paraná) regions of Brazil. Additionally, 764 sera from individuals with unrelated diseases were included to evaluate cross-reactivity. The unrelated diseases included COVID-19 (*n* = 10), dengue (*n* = 21), schistosomiasis (*n* = 45), filariasis (n = 50), hepatitis B (*n* = 78), hepatitis C (*n* = 49), HIV-1/2 (*n* = 76), HTLV-1/2 (*n* = 43), leprosy (*n* = 15), leptospirosis (*n* = 97), rubella (*n* = 16), syphilis (*n* = 110), and both cutaneous (*n* = 65) and visceral (*n* = 89) leishmaniasis.

### 3.2 Diagnostic performance of rTcD3 and rTcD6 IgG-ELISA

**Figure 3:**
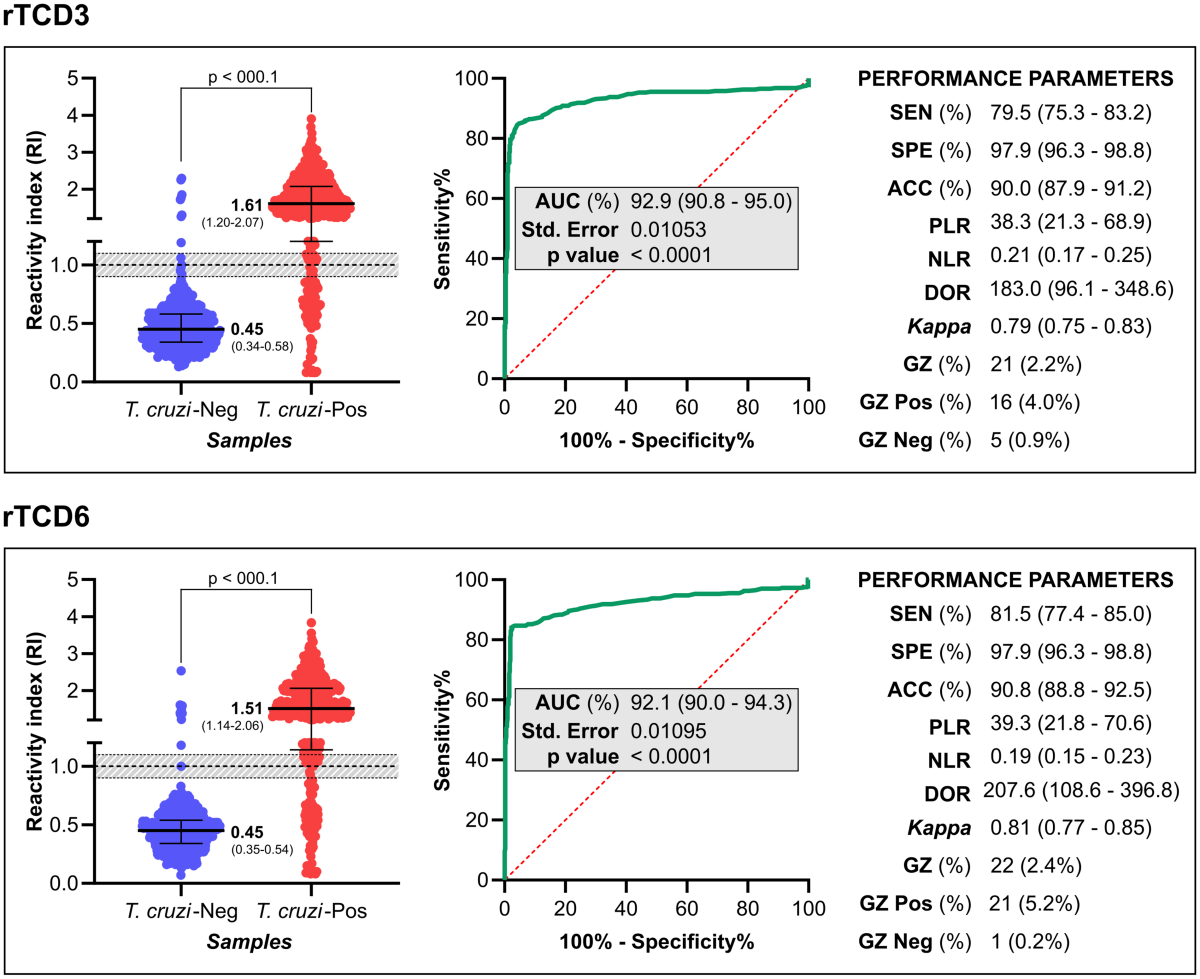
Diagnostic evaluation of the rTcD3 and rTcD6 proteins. The cut-off was set at 1.0 (dashed line), with an inconclusive range defined as RI = 1.0 ± 0.1 (shaded in grey). Each data point represents an individual sample, with box-and-whisker plots indicating the median and interquartile range. Statistical differences between groups were evaluated using the non-parametric Wilcoxon test, with significance levels (*p-values*) indicated in the Figure. Performance metrics for each antigen are detailed. *T. cruzi-*Pos, positive samples for *T. cruzi* infection; *T. cruzi-*Neg, negative samples for *T. cruzi* infection; ROC, receiver operating characteristic; AUC, Area Under Curve; SEN, sensitivity; SPE, specificity; ACC, accuracy; PLR, positive likelihood ratio; NLR, negative likelihood ratio; DOR, diagnostic odds ratio; Kappa, Cohen’s Kappa coefficient; CI, confidence interval; GZ, grey zone.

The RI values and performance metrics for the ELISA using rTcD3 and rTcD6 are presented in Figure 3 (individual data in Supplementary Table S1). Both recombinant proteins exhibited excellent discrimination between *T. cruzi-*infected and non-infected individuals, as reflected by high AUC values.

For the 405 positive samples, the sensitivity was 79.5% (95% CI: 75.3–83.2%) for rTcD3 and 81.5% (95% CI: 77.4–85.0%) for rTcD6. Both proteins achieved a specificity of 97.9% (95% CI: 96.3–98.8%). The highest accuracy was observed with rTcD6 (90.8, 95%CI: 88.8–92.5%), slightly outperforming rTcD3 (90.0, 95% CI: 87.9–91.2%).

The positive likelihood ratios (LR+) for rTcD3 and rTcD6 indicated a substantial increase in the probability of a positive result in CCD-positive individuals (LR+ > 10). Conversely, the negative likelihood ratios (LR−) were low (LR − < 0.2), signifying a reduced probability of false negatives in infected individuals.

The diagnostic odds ratio (DOR), integrating sensitivity, specificity, and likelihood ratios, was 183.0 for rTcD3 and 207.6 for rTcD6. Cohen’s kappa index (*κ*) indicated substantial agreement for rTcD3 (κ = 0.79) and almost perfect agreement for rTcD6 (κ = 0.81) compared with reference tests.

For *T. cruzi*-positive samples, the highest median RI was observed for rTcD3 (1.61; IQR 1.20–2.077), followed by rTcD6 (1.51; IQR 1.14–2.06). In contrast, *T. cruzi*-negative samples exhibited low median RI values for both antigens: 0.45 (IQR 0.34–0.58) for rTcD3 and 0.45 (IQR 0.35–0.54) for rTcD6.

### 3.3 Accuracy of rTcD3 and rTcD6 IgG-ELISA employing multiple testing algorithms

To minimize diagnosis uncertainty, serial and parallel testing approaches were applied based on the individual performance of the rTcD3 and rTcD6 proteins (Cebul et al., 1982). The serial testing approach resulted in a consistent decrease in sensitivity compared to individual protein testing or the parallel approach. However, specificity values were nearly 100% in this scheme (Table 1).

**Table 1:** Diagnostic performance of rTcD3 and rTcD6 IgG-ELISA, individually and in combination, using serial and parallel testing approaches.

Antigen	Approach	Sensitivity (%)	Specificity (%)	Accuracy (%)
rTcD3	Individual	79.5 CI 95% [75.3–83.2]	97.9 CI 95% [96.3–98.8]	90.0 CI 95% [87.9–91.2]
rTcD6	Individual	81.5 CI 95% [77.4–85.0]	97.9 CI 95% [96.3–98.8]	90.8 CI 95% [88.8–92.5]
rTcD3 + rTcD6	Series	64.8 CI 95% [58.3–70.6]	99.9 CI 95% [99.8–100]	84.7 CI 95% [81.9–87.3]
rTcD3 + rTcD6	Parallel	96.2 CI 95% [94.4–97.5]	95.9 CI 95% [92.8–97.7]	96.0 CI 95% [93.5–97.6]

In contrast, the parallel testing approach significantly improved sensitivity, achieving values exceeding 96.0%, along with a high true negative rate of 95.9%. The overall accuracy of the serial approach (84.7%) was lower compared to the individual protein tests and parallel approach, the latter of which demonstrated the highest accuracy (96.0%; Table 1).

### 3.4 Assessment of cross-reactivity with other infections

**Figure 4:**
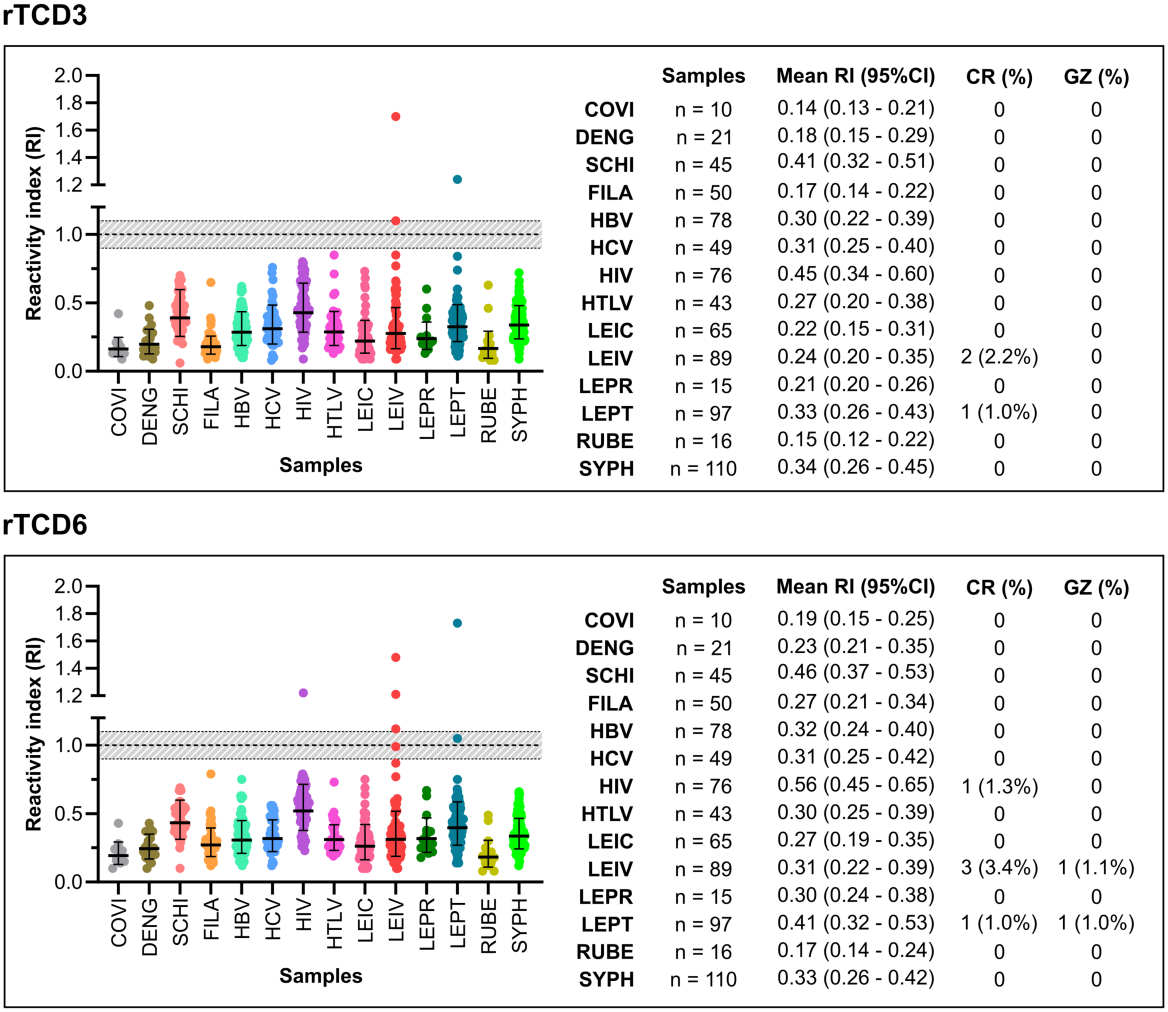
Cross-reactivity analysis of rTcD3 and rTcD6 proteins. The cut-off was set at 1.0 (dashed line). Samples with a Reactivity Index (RI) above 1.0 were considered positive, while samples with an RI below 1.0 were considered negative. An inconclusive range was defined as RI = 1.0 ± 0.1 (shaded in grey). False-positive results are defined as samples from individuals with other infections that tested positive in the rTcD3 or rTcD6 ELISA. Each data point represents an individual sample. Box-and-whisker plots show the median and interquartile range of RI values for each infection group. COVI, COVID-19; DENG, dengue; SCHI, schistosomiasis; FILA, filariasis; HBV, hepatitis B virus; HCV, hepatitis C virus; HIV, human immunodeficiency virus; HTLV, Human T-lymphotropic virus 1; LEIC, cutaneous leishmaniasis; LEIV, visceral leishmaniasis; LEPR, leprosy; LEPT, leptospirosis; RUBE, rubella; SYPH, syphilis; CI, confidence interval; CR, cross-reactivity; GR, grey zone.

The rTcD3 and rTcD6 IgG-ELISA were evaluated for cross-reactivity using sera from individuals infected with bacteria, virus and non-*T. cruzi* parasites (Figure 4; Supplementary Table S2). False-positive results for rTcD3 were observed in one leptospirosis-positive sample and two leishmaniasis-positive samples. For rTcD6, false positives were recorded in one leptospirosis-positive sample, three leishmaniasis-positive samples and one HIV-positive individual. Within the inconclusive range (RI = 1.0 ± 0.1), one leptospirosis-positive and one leishmania-positive sample yielded an inconclusive result for rTcD6.

## 4 Discussion

Developing a reliable reference test for diagnosing CCD remains a priority on the WHO’s agenda. The current necessity for performing multiple tests—often two or three in cases of discordant results—using diverse assay formats and antigen sources imposes substantial financial and logistical burdens. These challenges are particularly significant in resource-limited endemic regions or emergency room settings, where infected individuals may assist without prior diagnosis. Numerous recombinant protein-based ELISA assays for the diagnosis of CCD have been developed and documented, exhibiting specificity ranging from 93.8 to 100.0%, and sensitivity spanning 65.0 to 100.0%, contingent on the specific antigen employed and the testing conditions (Resende et al., 2024). Among the most widely studied antigens, proteins such as B13, 1F8, JL7, KMP11, FRA, and CRA have demonstrated promising diagnostic performance and have consequently been integrated into several commercial diagnostic kits. Despite these developments, a universal gold-standard antigen for Chagas disease diagnosis has not yet been established. A major limitation is the high variability in serological panels across studies, the diverse geographic origins of the tested populations, and the use of different laboratory methodologies, which complicate direct comparisons among assays. Furthermore, cross-reactivity with other infectious diseases remains a critical concern, particularly in regions where *T. cruzi* and *Leishmania* spp. co-exist (Resende et al., 2024). For example, a comparative study evaluating four commercial IgG *T. cruzi* enzyme immunoassays using 1,433 serum samples from Brazil reported for Gold ELISA Chagas (Rem) 100% sensitivity and 100% specificity; for Imuno-ELISA Chagas (Wama Diagnóstica) 99.5% sensitivity with 99.2% specificity; for ELISA Chagas III (BIOSChile) 97.3% sensitivity with 100% specificity, and for Pathozyme® Chagas (Omega) 99.5% sensitivity with 97.0% specificity (Santos et al., 2016). However, these assays exhibited cross-reactivity with pathogens such as rubella virus, measles virus, syphilis, hepatitis C virus, and, most notably, *Leishmania* spp., with values ranging from 8.6 to 42.9%. A previous study showed that Pathozyme® Chagas (Omega) yields 75% sensitivity and 100% specificity while Chagatest Rec v3.0 (Wiener) yields 81.5% sensitivity and 100% specificity when tested against serum samples from Brazilian individuals infected with *T. cruzi* and *Leishmania* sp. (Caballero et al., 2007). Such findings highlight the importance of evaluating diagnostic tools not only based on their analytical performance but also on their ability to distinguish *T. cruzi* infection from other common diseases in endemic regions. In addition to these concerns, it is important to note that many existing studies do not include sera from countries such as Mexico, where a high prevalence of false-negative results has been reported. This is likely due to differences in the circulating discrete typing units (DTUs) of *T. cruzi* and variations in host immune responses (Whitman et al., 2019; Kelly et al., 2021; Truyens et al., 2021). Consequently, despite decades of research, no single serological assay has achieved perfect sensitivity and specificity, largely due to the genetic diversity of the parasite and the heterogeneity of host antibody repertoires across endemic regions.

In this context, we recently proposed two recombinant proteins, rTcD3 and rTcD6, as potential diagnostic candidates in ELISA format. The proteins are derived from distinct regions of the hypothetical *T. cruzi* protein Tc323, with rTcD3 corresponding to an internal domain and rTcD6 to its C-terminal region. In a prior study using 333 CCD-positive and 141 CCD-negative samples from seven endemic countries across Latin America, rTcD3 exhibited sensitivity and specificity values of 90.7 and 92.2%, respectively, while rTcD6 demonstrated values of 93.1 and 93.6% (Ossowski et al., 2024). These results highlight the potential of both antigens to reliably distinguish *T. cruzi*-infected individuals from non-infected individuals, irrespective of parasite strain or host antibody variability.

This robust performance can be attributed to the conserved nature of Tc323 across parasite DTUs, with limited cross-reactivity restricted to non-pathogenic trypanosomatids (*Trypanosoma rangeli* and *Paratrypanosoma*) and absent in other eukaryotes outside the kinetoplastids, including mammalian hosts parasitized by *T. cruzi*. Sequence alignment using the MUSCLE algorithm confirmed that TcD3 and TcD6 exhibit >97% sequence conservation across different parasite lineages. Additionally, both recombinant proteins were effective in diagnosing CCD with and without cardiac manifestations, underscoring their diagnostic utility, though they do not provide prognostic information on disease severity (Ossowski et al., 2024).

In the present study, we evaluated the diagnostic performance of these proteins in a larger number of individuals from Brazil, a country where TcII is prevalent (Zingales and Macedo, 2023). Specificity values for both antigens were consistently high (97.9%), but sensitivity compared to our previous work decreased to 79.5 and 81.5% for rTcD3 and rTcD6, respectively. The increase in specificity is particularly noteworthy, as a higher number of *T. cruzi*-negative samples were included in this analysis. Conversely, the decrease in sensitivity can primarily be attributed to a difference in false-negative results, which rose from 31 to 83 and 19 to 75 samples to rTcD3 and rTcD6, respectively (Supplementary Figure S1). In our opinion, as Tc323 domains are conserved across all parasite strains available in the database, the sensitivity reduction may reflect variations in antibody response mounted by individuals in the area. It is widely documented, particularly in response to vaccines, that host genetic variations—such as HLA class I and II alleles, and single-nucleotide polymorphisms in coding and noncoding regions of cytokines and their receptors—can contribute to differences in antibody response among countries. Furthermore, intrinsic factors such as age and sex, as well as non-genetic factors like smoking, diet, medical and vaccination history also influence the human antibody repertoire against pathogens (Meyer et al., 2021; Truyens et al., 2021; Olin et al., 2023). To improve diagnostic performance, a chimeric protein combining TcD3 and TcD6 could present additional antigenic determinants or a more favorable tertiary structure, enhancing antibody binding following plate immobilization.

Cross-reactivity testing with 764 samples from individuals infected with other parasites, viruses, and bacteria demonstrated minimal interference. Only one leptospirosis-positive sample showed reactivity with both proteins. Additionally, two visceral leishmaniasis-positive samples yielded false positives for rTcD3, while three of these, along with one HIV-positive sample, tested positive for rTcD6. These findings align with prior observations of minimal cross-reactivity with toxoplasmosis-positive sera, indicating that IgG-ELISAs exhibit excellent specificity (<0.9% cross-reactivity).

Although the specificity of the rTcD3 and rTcD6 ELISA assays is comparable to that of currently available commercial tests, their individual sensitivities remain slightly lower than those reported for the highest-performing assays (Resende et al., 2024). In the context of serological diagnosis, the World Health Organization (WHO) currently recommends the use of two concordant serological tests, followed by a third assay in cases of discordant results, to enhance diagnostic accuracy. In our study, the performance of rTcD3 and rTcD6 when used individually suggests that neither assay alone would be sufficient to replace the current diagnostic algorithm, primarily due to their marginally reduced sensitivity. However, when applied in a parallel testing strategy, the combination of both assays resulted in a marked improvement in diagnostic performance, yielding a sensitivity exceeding 96.0% and a high true negative rate of 95.9%. These findings underscore the potential of combining complementary antigen targets to enhance diagnostic yield. Building upon this observation, we are currently developing a chimeric protein that incorporates immunodominant regions of both TcD3 and TcD6, aiming to replicate the advantages of parallel testing within a single, streamlined assay. We anticipate that further optimization and validation of this approach may offer a more practical, cost-effective, and accurate serological tool for the diagnosis of Chagas disease, particularly in resource-limited settings.

## 5 Conclusion

In sum, this study demonstrates that rTcD3 and rTcD6-based IgG-ELISAs are valuable and sensitive diagnostic tools for detecting chronic *T. cruzi* infection across a broad spectrum of both endemic and non-endemic regions of Brazil. These assays exhibit specificity against antibodies generated in response to other pathogens, including closely related *Leishmania* species. Moreover, the results emphasize the promising potential of utilizing hypothetical proteins for high-specificity diagnostics, thus providing a solid foundation for further exploration of alternative antigens for CCD diagnosis. Future research should prioritize larger sample sizes, validation within natural populations, and an assessment of these assays’ applicability for post-treatment monitoring and the differentiation between acute and chronic infections.

## 6 Limitations

This study is not without its limitations. Notably, the exclusion of samples with discordant results from commercial assays, which were retested, may have led to an overestimation of specificity. Additionally, the study’s geographic scope was limited to specific regions of Brazil, which may impact the generalizability of the findings. Furthermore, a comparative analysis with existing diagnostic methodologies, particularly regarding factors such as cost-effectiveness, time efficiency, and accessibility, was not performed.

Another limitation is the lack of information on the specific *T. cruzi* DTUs infecting each individual, and the absence of data characterizing the reactivity of the positive sera against specific DTUs. While our results suggest that the rTcD3 and rTcD6 antigens are capable of eliciting a consistent antibody response across a diverse population, further studies are needed to confirm this finding and to investigate the potential impact of DTU-specific variations on the diagnostic performance of our assay.

## Data Availability

The original contributions presented in the study are included in the article/Supplementary material, further inquiries can be directed to the corresponding authors.

## Supplementary material

Data Sheet 1 = S1 Checklist.

Supplementary Table 1 = Supplementary Table S1.

Supplementary Table 2 = Supplementary Table S2.

Image 1 = Supplementary Figure S1.

SUPPLEMENTARY FIGURE S1 UpSet plot illustrating the intersections of reactivity to rTcD3 and/or rTcD6. The bar plot displays the count of seroreactive positive **(A)** or negative **(B)** samples for each protein, with green indicating samples recognizing only rTcD6, violet for rTcD3, and sky blue for samples recognizing either antigen. Shared reactivity between both antigens is depicted in grey. The bar plots on the left show the total number of samples tested.
